# Supplementary material for: Mimicry of emergent traits amplifies coastal restoration success
Source: Nat Commun. 2020 Jul 22;11:3668. doi: 10.1038/s41467-020-17438-4 (PMC7376209; doi:10.1038/s41467-020-17438-4)
Supplement: Supplementary file 2 — Supplementary Information [file 41467_2020_17438_MOESM2_ESM.pdf]

Supplementary Information

Mimicry of emergent traits amplifies coastal restoration success

Ralph J.M. Temmink*, Marjolijn J.A. Christianen*, Gregory S. Fivash*, Christine Angelini, Christoffer Boström, Karin Didderen, Sabine M. Engel, Nicole Esteban, Jeffrey L. Gaeckle, Karine Gagnon, Laura L. Govers, Eduardo Infantes, Marieke M. van Katwijk, Silvija Kipson, Leon P.M. Lamers, Wouter Lengkeek, Brian R. Silliman, Brigitta I. van Tussenbroek, Richard K.F. Unsworth, Siti Maryam Yaakub, Tjeerd, J. Bouma, Tjisse van der Heide

*these authors contributed equally to this paper

Correspondence to: tjisse.van.der.heide@nioz.nl, r.temmink@science.ru.nl

Index:

Supplementary Figures 1 to 2
Supplementary Tables 1 to 3

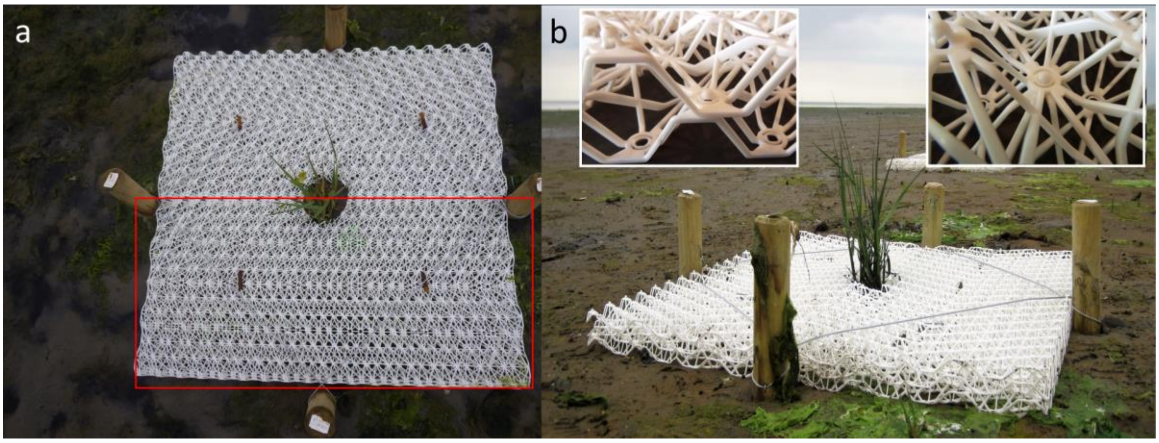

Supplementary Figure 1. Example of a plot and material close-ups. (**A**) Overview of a 91x91x6 cm (L x W x H) cordgrass plot in the Netherlands, consisting of two adjacently placed sections/modules (indicated by the red rectangle), that each consist of 3 stacked 91x45.5x2 cm sheets. Half a circle was cut out from the centre along longest side of each module to create a 10 cm diameter transplant opening in the middle of the plot. (**B**) A sideview of a plot, with inserts detailing the structure's 3D-complexity (left) and clicking mechanism (right) to connect sheets. Photos: RJMT.

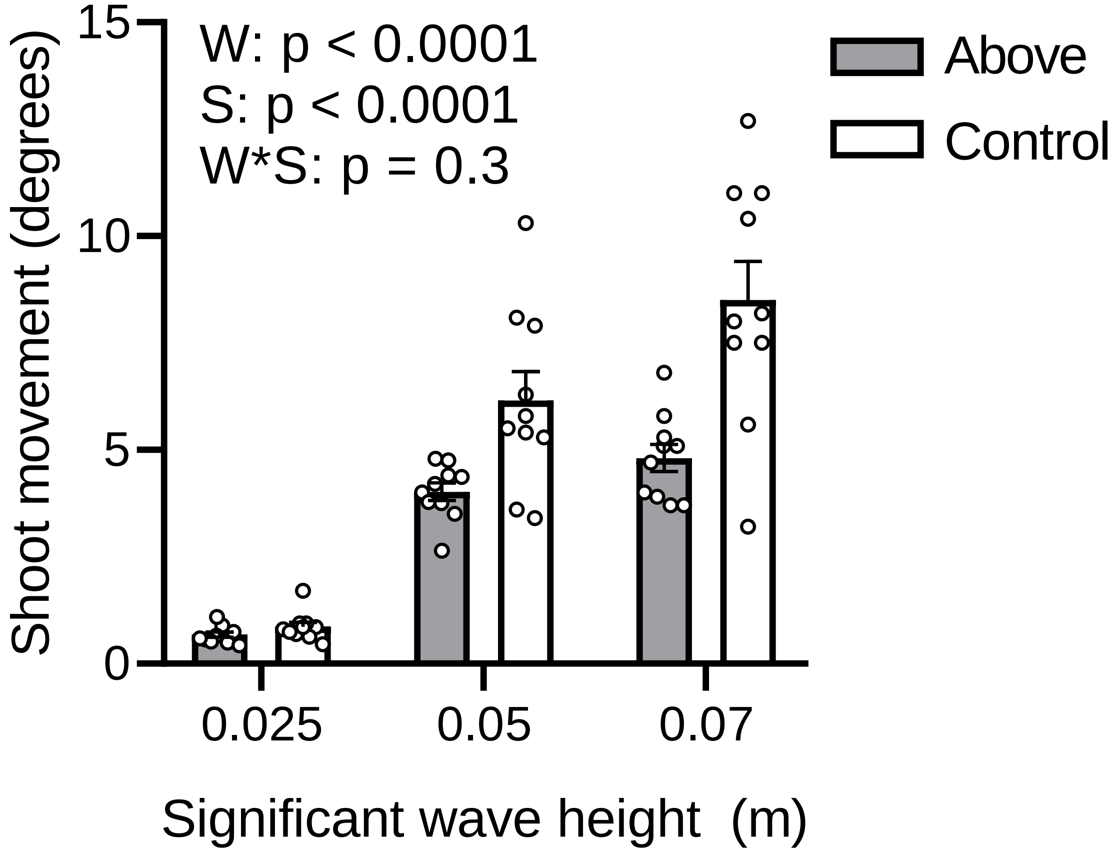

Supplementary Figure 2. Cordgrass stem movement with and without aboveground establishment structures. Cordgrass stem mimics situated within the centre of an aboveground establishment structure (Above, grey bars) or on bare sediment (Control, white bars). Data are presented as mean values + SEM (n = 10). Results of GLM on log-transformed data for main (W: wave intensity, S: structure) and interactive (W*S) effects are shown with p-values. Exact p-values are shown when p > 0.0001 (two-sided). Source data are provided as a Source Data file.

Supplementary Table 1. Site coordinates and hydrological conditions.

**Species**	**Site** (Country)	**Latitude** Degrees	**Longitude** Degrees	**Experimental period** Months (Year)	**Depth** (m) | **Inundation percentage** (%)	**Maximum fetch length direction**	**Range highest fetch** (km)	**Wind speed** (m/s)*	**Significant wave height** (m)**	**Wave formation limiting factor**
**Temperate**										
Zostera marina	Gullmarsfjord (Sweden)	58.335320	11.542482	14 (2017-2018)	1.3 | 100	North-east to South-east	2-4	11.3[(c)]	0.26-0.36[(g)]	Fetch length
Spartina anglica	De Schorren (the Netherlands)	53.123817	4.900338	16 (2016-2017)	0.24[a] | 20	North-East to South	20-80	13.7[(d)]	0.12-0.57[(g, **)]	Water depth
**(sub)Tropical**										
Thalassia testudinum	Lac Bay (Bonaire)	12.097624	-68.230259	22 (2017-2018)	1.6 | 100	North-East to South-east	0.2-0.4	11.4[(e)]	0.08-0.1[(g)]	Fetch length
Spartina alterniflora	Marineland (Florida, USA)	29.683471	-81.224263	12 (2018-2019)	0.42[b] | 40	South to South-east	0.6-1.2	11.8[(f)]	0.15-0.2[(g)], 0.1-0.4[(h)]	Fetch length

* The average of the top 5% strongest winds from the direction of the highest fetch length.
** We calculated the significant wave height based on the 33% highest water levels during the experiment (95 cm).
[a]https://waterinfo.rws.nl, station Oudeschild, [b]https://tidesandcurrents.noaa.gov, station Crescent Beach, [c]https://www.weather.loven.gu.se/kristineberg/en/, [d]KNMI, station Vlieland, [e]https://rp5.ru/, station Kralendijk Airport, [f]https://www.ndbc.noaa.gov/, station St. Augustine, [g]https://planetcalc.com. [h]square root average and maximum height of boat-induced waves[1].

Supplementary Table 2. Cost scenarios for emergent trait-based restoration of salt marshes and seagrasses. The costs (in US$) of four restoration scenarios are calculated based on a patch-wise initial transplant cover that restoration practitioners may select assuming two contrasting lateral extension rates of transplants (i.e., fast *vs.* slow growth), and two potential restoration periods in which complete recovery should be accomplished; i.e. 5 (short) *vs.* 10 (long) years to establish a continuous vegetation stand. The chosen restoration periods and designs are meant to illustrate how different choices affect the costs. In each scenario, the required initial cover by emergent traits mimics (% of a hectare, or plots per hectare) differs depending on the selected restoration period and plant species. See methods for details.

**Salt marsh**							
Scenario 1: short time, fast growth				Scenario 2: long time, fast growth			
Initial cover (%)	Plots (# ha^{-1})[a,1]	Costs ($ ha^{-1})	Category	Initial cover (%)	Plots (# ha^{-1})[a,1]	Costs ($ ha^{-1})	Category
6	625	15,000	BESE[b]	2	156	3,750	BESE[b]
		5,000	Rebars[c]			1,250	Rebars[c]
		4,375	Labor[d]			1,094	Labor[d]
		625	Transplants[e]			156	Transplants[e]
		**25,000**	**Total**			**6,250**	**Total**
Scenario 3: short time, slow growth				Scenario 4: long time, slow growth			
Initial cover (%)	Plots (# ha^{-1})[a,2]	Costs ($)	Category	Initial cover (%)	Plots (# ha^{-1})[a,2]	Costs ($ ha^{-1})	Category
25	2500	60,000	BESE[b]	6	625	15,000	BESE[b]
		20,000	Rebars[c]			5,000	Rebars[c]
		17,500	Labor[d]			4,375	Labor[d]
		2,500	Transplants[e]			625	Transplants[e]
		**100,000**	**Total**			**25,000**	**Total**
**Seagrass**							
Scenario 1: short time, fast growth				Scenario 2: long time, fast growth			
Initial cover (%)	Plots (# ha^{-1})[a,3]	Costs ($)	Category	Initial cover (%)	Plots (# ha^{-1})[a,3]	Costs ($ ha^{-1})	Category
3	278	6,670	BESE[b]	1	69	1,670	BESE[b]
		2,220	Rebars[c]			560	Rebars[c]
		10,700	Labor[d]			2,700	Labor[d]
		280	Transplants[e]			69	Transplants[e]
		**19,870**	**Total**			**4,999**	**Total**
Scenario 3: short time, slow growth				Scenario 4: long time, slow growth			
Initial cover (%)	Plots (# ha^{-1})[a,4]	Costs ($ ha^{-1})	Category	Initial cover (%)	Plots (# ha^{-1})[a,4]	Costs ($ ha^{-1})	Category
39	3906	93,750	BESE[b]	10	977	23,440	BESE[b]
		31,250	Rebars[c]			7,810	Rebars[c]
		150,400	Labor[d]			37,600	Labor[d]
		3,900	Transplants[e]			980	Transplants[e]
		**279,300**	**Total**			**69,830**	**Total**

[a:] Spread equally through space. Plots as described in the methods. Based on plot diameter after 5 (short time) or 10 years (long time) of lateral growth.
[b:] 3 sheets thick (dimension sheet 92x45.5x2.0 cm), 2 blocks of 3 sheets, 4 dollar per sheet.
[c:] 8 rebar anchors per plot, 1 dollar per rebar.
[d:] Construction time plus organisation/project planning time (0.5 + 0.2 h / plot for belowground, 0.1 + 0.1 h / plot for aboveground structures) at an hourly rate of 35 US$. Scuba, required to construct subtidal seagrass plots, include $10/h for boat fees and $10/h for compressed air (assuming equipment is owned).
[e:] Transplants of 10*10cm (1 dollar per transplant).

[1:] Based on a lateral expansion rate of 0.4 m/year ([2,3])
[2:] Based on a lateral expansion rate of 0.2 m/year ([2])
[3:] Based on a lateral expansion rate of 0.6 m/year ([4]). This is a conservative estimate of fast growth as some species have a lateral expansion rate greater than 100 cm/year.
[4:] Based on a lateral expansion rate of 0.16 m/year ([5])

Supplementary Table 3. Summary of all statistical results. Numbers indicate the statistical test used: $^{(1)}$ = GLM with binomial distribution, $^{(2)}$ = GLMM with Poisson distribution and block effect, $^{(2*)}$ = GLM with a negative binomial distribution, $^{(3)}$ = Kruskal-Wallis, $^{(4)}$ = t-test with unequal variances, and $^{(5)}$ = LMM with block effect. Exact p-values are shown if $p >$ 0.0001.

**Treatment**	**Variable**	***df***	***Chi-$^{(2)}$, t-$^{(4)}$ or F-value$^{(5)}$***	***p-value***
**The Netherlands, temperate, *Spartina anglica***				
Aboveground	Survival[1]	2	22.17	<0.0001
Belowground				
Control				
**The Netherlands, temperate, *Spartina anglica***				
Aboveground	Shoot number[2]	2	684.56	<0.0001
Belowground				
Control				
**The Netherlands, temperate, *Spartina anglica***				
Aboveground	Maximum lateral expansion[3]	2	7.69	0.02
Belowground				
Control				
**Florida, USA, subtropical, *Spartina alterniflora***				
Aboveground	Survival[1]	2	15.28	0.0005
Belowground				
Control				
**Florida USA, subtropical, *Spartina alterniflora***				
Aboveground	Shoot number[2]	2	48.03	<0.0001
Belowground				
Control				
**Florida USA, subtropical, *Spartina alterniflora***				
Aboveground	Maximum lateral expansion[3]	2	10.57	0.001
Belowground				
Control				
**Sweden, temperate, *Zostera marina***				
Aboveground	Survival[1]	2	6.28	0.04
Belowground				
Control				
**Sweden, temperate, *Zostera marina***				
Aboveground	Shoot number[2*]	2	33.3	<0.0001
Belowground				
Control				
**Sweden, temperate, *Zostera marina***				
Aboveground	Maximum lateral expansion[3]	2	9.37	0.01
Belowground				
Control				
**Bonaire, tropical, *Thalassia testudinum***				
Aboveground	Survival[1]	2	6.28	0.04
Belowground				
Control				

**Bonaire, tropical, *Thalassia testudinum***				
Aboveground	Shoot number[2]	2	28.01	<0.0001
Belowground				
Control				
**Bonaire, tropical, *Thalassia testudinum***				
Aboveground	Maximum lateral expansion[3]	2	8.64	0.02
Belowground				
Control				
**Wave flume experiment with cordgrass mimics**				
Aboveground	Shoot movement[4]	1	-3.8758	0.002
Control				
**Seagrass sites (sediment movement)**[5]				
Structure	Sediment movement[5]	2.1	22.393	<0.0001
Location		1.6	24.015	0.003

Supplementary references

1 Herbert, D. *et al.* Mitigating erosional effects induced by boat wakes with living shorelines. *Sustainability* **10**, 436 (2018).

2 Silliman, B. R. *et al.* Facilitation shifts paradigms and can amplify coastal restoration efforts. *Proceedings of the National Academy of Sciences* **112**, 14295-14300, doi:10.1073/pnas.1515297112 (2015).

3 Schwarz, C. *et al.* Abiotic factors governing the establishment and expansion of two salt marsh plants in the Yangtze Estuary, China. *Wetlands* **31**, 1011-1021 (2011).

4 Marbà, N. & Duarte, C. M. Rhizome elongation and seagrass clonal growth. *Marine Ecology Progress Series* **174**, 269-280 (1998).

5 Bastyan, G. R. & Cambridge, M. L. Transplantation as a method for restoring the seagrass Posidonia australis. *Estuarine, Coastal and Shelf Science* **79**, 289-299.
