# Supplementary material for: Mimicry of emergent traits amplifies coastal restoration success
Source: Nat Commun. 2020 Jul 22;11:3668. doi: 10.1038/s41467-020-17438-4 (PMC7376209; doi:10.1038/s41467-020-17438-4)
Supplement: Supplementary file 3 — Reporting Summary [file 41467_2020_17438_MOESM3_ESM.pdf]

nature research

Corresponding author(s): Tjisse van de Heide, Ralph J.M. Temmink

Last updated by author(s): Jun 15, 2020

Reporting Summary

Nature Research wishes to improve the reproducibility of the work that we publish. This form provides structure for consistency and transparency in reporting. For further information on Nature Research policies, see Authors & Referees and the Editorial Policy Checklist.

Statistics

For all statistical analyses, confirm that the following items are present in the figure legend, table legend, main text, or Methods section.

n/a	Confirmed	
☐	☒	The exact sample size (n) for each experimental group/condition, given as a discrete number and unit of measurement
☐	☒	A statement on whether measurements were taken from distinct samples or whether the same sample was measured repeatedly
☐	☒	The statistical test(s) used AND whether they are one- or two-sided<br>*Only common tests should be described solely by name; describe more complex techniques in the Methods section.*
☒	☐	A description of all covariates tested
☐	☒	A description of any assumptions or corrections, such as tests of normality and adjustment for multiple comparisons
☐	☒	A full description of the statistical parameters including central tendency (e.g. means) or other basic estimates (e.g. regression coefficient) AND variation (e.g. standard deviation) or associated estimates of uncertainty (e.g. confidence intervals)
☐	☒	For null hypothesis testing, the test statistic (e.g. F, t, r) with confidence intervals, effect sizes, degrees of freedom and P value noted<br>*Give P values as exact values whenever suitable.*
☒	☐	For Bayesian analysis, information on the choice of priors and Markov chain Monte Carlo settings
☒	☐	For hierarchical and complex designs, identification of the appropriate level for tests and full reporting of outcomes
☒	☐	Estimates of effect sizes (e.g. Cohen's d, Pearson's r), indicating how they were calculated

Our web collection on statistics for biologists contains articles on many of the points above.

Software and code

Policy information about availability of computer code

Data collection	There was no software used for data collection.
Data analysis	R version 3.6.0 with packages lme4 and lsmeans for experimental comparisons.<br>ImageJ for photo analyses.

For manuscripts utilizing custom algorithms or software that are central to the research but not yet described in published literature, software must be made available to editors/reviewers. We strongly encourage code deposition in a community repository (e.g. GitHub). See the Nature Research guidelines for submitting code & software for further information.

Data

Policy information about availability of data

All manuscripts must include a data availability statement. This statement should provide the following information, where applicable:

- Accession codes, unique identifiers, or web links for publicly available datasets
- A list of figures that have associated raw data
- A description of any restrictions on data availability

Figures 2, 3, 4, 5, S2, and Tables S1, S2 are associated with raw empirical data. All data that support the main findings of this study are available via the Data Archiving and Networked Services (DANS) EASY (https://doi.org/10.17026/dans-xx2-s4c6).

Field-specific reporting

Please select the one below that is the best fit for your research. If you are not sure, read the appropriate sections before making your selection.

☐ Life sciences ☐ Behavioural & social sciences ☒ Ecological, evolutionary & environmental sciences

For a reference copy of the document with all sections, see nature.com/documents/nr-reporting-summary-flat.pdf

Ecological, evolutionary & environmental sciences study design

All studies must disclose on these points even when the disclosure is negative.

Study description	We tested the potential of using trait-based biomimicry as a general restoration approach by conducting experiments with generally identical setups across two ecosystems and climate zones (tropical and temperate seagrass and salt marsh systems). More specifically, we carried out transplantation experiments with Zostera marina (temperate seagrass) in Sweden, Thalassia testudinum (tropical seagrass) in Bonaire, Spartina anglica in (temperate salt marsh) the Netherlands, and Spartina alterniflora (tropical salt marsh) in Florida, USA. At each of these experimental locations, vegetation was previously mapped, but had disappeared. At each location, transplants were planted in the center of control plots, or in plots with below- or aboveground establishment structures, respectively, yielding a total of 3 treatments. Belowground establishment structures were buried in the sediment to simulate dense seagrass- or cordgrass root mats. Aboveground structures were placed on the sediment surface to simulate dense patches of stiff (i.e. cordgrass-like) vegetation stems. Plots were contructed using a randomized block design, with 7 replicate blocks in the Netherlands, 8 in Florida, and 4 in Bonaire and Sweden. At the end of the experimental periods, transplant survival (%), shoot number (count) and maximum lateral expansion (cm) were determined in each of the four ecosystems. Experimental periods lasted 14 months in Sweden, 16 months in the Netherlands, 22 months in Bonaire, and 12 months in Florida).<br><br>Sediment movement was measured in the Bonaire and Sweden experiments by placing sediment-burial pins for one month in the center of each plot (n = 4 per treatment). A flat stainless steel ring was placed around the pin on the sediment surface, after which the distance between the upper tip of the pin and the sediment level was measured. During the measurement period, the ring moves downward each time the sediment become unstable. As a proxy of sediment mobility, we therefore measured the distance between the sediment level and the ring.<br><br>We used a wave flume to study how cordgrass stem movement was affected by the aboveground establishment structure. Within the test section, we placed 15 162-mm long cordgrass mimics, resembling natural cordgrass vegetation, fixed to a mesh in the 10 cm diameter opening of the aboveground establishment structure (dimensions: 90*60*6 cm (L*W*H)) or at a bare sediment control. Next, mimics were subjected to 25, 50 and 70 mm high waves, during which we measured stem movement of 10 stems.
Research sample	We transplanted Zostera marina (Sweden) and Thalassia testudinum (Bonaire, both seagrasses), Spartina anglica (the Netherlands) and Spartina alterniflora (US Florida, both cordgrasses) into bare controls, belowground and belowground establishment structures, respectively. We measured transplant survival, shoot number and lateral expansion at the end of the experimental period to quantify restoration success.
Sampling strategy	Transplant survival (%), shoot number (count) and maximum lateral expansion (cm) was determined at the end of the experimental periods in each of the four ecosystems. In the two seagrass systems, we also measured sediment movement at the center of each plot by placing sediment-burial pins over the course of one month. Using cordgrass mimics in a wave flume at NIOZ Yerseke, we determined how stem movement was affected by the aboveground establishment structure. Sample sizes were based on literature (e.g. earlier ecological (restoration) experiments), and the space and time available at the field sites.
Data collection	Transplant survival was determined by noting the presence or absence of vegetation in each plot, after which the number of shoots were counted. Lateral outgrowth was measured as the straight-line distance from the plot center to newest shoot at the end of the longest rhizome. All authors and several volunteers were involved in carrying out the field experiments. RJMT, GSF, KD recorded the data in the Netherlands; RJMT, WL, LPML, TvdH, CA, GSF in US Florida; MJAC and SME in Bonaire; and EI in Sweden.<br><br>Sediment movement was measured in the Bonaire and Sweden experiments by placing sediment-burial pins for a month in the center of each plot. Next, a flat ring was placed around the pin on the sediment surface, after which the distance between the upper tip of the pin and the sediment level was measured. Over the course of the following month, the ring moved downward each time the sediment became unstable. As a proxy of sediment mobility, we therefore measured the distance between the sediment level and the ring. MJAC and SME collected data in Bonaire, and EI in Sweden.<br><br>For cordgrass mimic stem movement, mimics were subjected to 25, 50 and 70 mm high waves, while stem movement was recorded from the side for 60 seconds by a video camera. RJMT and GSF and volunteers collected stem movement data.
Timing and spatial scale	The plot size of a single experimental unit was approximately 1m2. The experiment in the Netherlands ran from 6-2016 until 9-2017, in Florida from 4-2018 till 4-2019, in Bonaire from 2-2017 till 12-2018, and in Sweden from 6-2017 till 9-2018. Long-term effects of each treatment on the described variables were assessed at the end of the experimental period. Each experiment ran for at least a year to include all seasons.
Data exclusions	No data were excluded.
Reproducibility	The treatments were constructed in a randomized block design in all ecosystems.

Randomization	The treatments were set up in a randomized block design in all ecosystems.
Blinding	Treatments were set up randomly within each block. Once constructed, blinding was not applicable.

Did the study involve field work? ☒ Yes ☐ No

Field work, collection and transport

Field conditions	Fieldwork was performed during calm and dry days.
Location	We conducted the experiments in Sweden (temperate seagrass, Zostera marina, Gullmars Fjord, Latitude 58.335320, longitude 11.542482), Bonaire (tropical seagrass, Thalassia testudinum, Lac Bay, latitude 12.097624, longitude -68.230259), the Netherlands (temperate salt marsh, Spartina anglica, de Schorren, lonitude 53.123817, latitude 4.900338), and in Florida, USA (tropical salt marsh, Spartina alterniflora, Marineland, latitude 29.683471, longitude -81.224263).
Access and import/export	Prior to setting up the experiment, we obtained permits for site access, experimental manipulations, and collection of plant material from Natuurmonumenten (the Netherlands), Florida Department of Environmental Protection (US Florida), Openbaar Lichaam Bonaire (Bonaire) and Länsstyrelsen Västra Gotaland (Sweden).
Disturbance	No disturbance beyond the collection of transplant material. We obtained this material from healthy and large vegetation stands to minimize the disturbance on the ecosystem.

Reporting for specific materials, systems and methods

We require information from authors about some types of materials, experimental systems and methods used in many studies. Here, indicate whether each material, system or method listed is relevant to your study. If you are not sure if a list item applies to your research, read the appropriate section before selecting a response.

Materials & experimental systems

n/a	Involved in the study
☒	☐ Antibodies
☒	☐ Eukaryotic cell lines
☒	☐ Palaeontology
☒	☐ Animals and other organisms
☒	☐ Human research participants
☒	☐ Clinical data

Methods

n/a	Involved in the study
☒	☐ ChIP-seq
☒	☐ Flow cytometry
☒	☐ MRI-based neuroimaging
